# Targeted elastin-like polypeptide fusion protein for near-infrared imaging of human and canine urothelial carcinoma

**DOI:** 10.18632/oncotarget.28271

**Published:** 2022-09-06

**Authors:** Aayush Aayush, Saloni Darji, Deepika Dhawan, Alexander Enstrom, Meaghan M. Broman, Muhammad T. Idrees, Hristos Kaimakliotis, Timothy Ratliff, Deborah Knapp, David Thompson

**Affiliations:** ^1^Department of Chemistry, Purdue University, Bindley Bioscience Center, West Lafayette, IN 47907, USA; ^2^Department of Veterinary Clinical Sciences, Purdue University, West Lafayette, IN 47907, USA; ^3^Purdue University Center for Cancer Research, Purdue University, West Lafayette, IN 47907, USA; ^4^Department of Comparative Pathobiology, Purdue University, West Lafayette, IN 47907, USA; ^5^Department of Pathology and Laboratory Medicine, Indiana University, Indianapolis, IN 46202, USA; ^6^Department of Urology, Indiana University School of Medicine, Indianapolis, IN 46202, USA; ^*^These authors contributed equally to this work

**Keywords:** bladder cancer, elastin-like polypeptide, NIR imaging, epidermal growth factor receptor (EGFR), translational studies

## Abstract

Cystoscopic visualization of bladder cancer is an essential method for initial bladder cancer detection and diagnosis, transurethral resection, and monitoring for recurrence. We sought to develop a new intravesical imaging agent that is more specific and sensitive using a polypeptide based NIR (near-infrared) probe designed to detect cells bearing epidermal growth factor receptors (EGFR) that are overexpressed in 80% of urothelial carcinoma (UC) cases. The NIR imaging agent consisted of an elastin like polypeptide (ELP) fused with epidermal growth factor (EGF) and conjugated to Cy5.5 to give Cy5.5-N24-EGF as a NIR contrast agent. In addition to evaluation in human cells and tissues, the agent was tested in canine cell lines and tissue samples with naturally occurring invasive UC. Flow cytometry and confocal microscopy were used to test cell-associated fluorescence of the probe in T24 human UC cells, and in K9TCC-SH (high EGFR expression) and K9TCC-Original (low EGF expression) canine cell lines. The probe specifically engages these cells through EGFR within 15 min of incubation and reached saturation within a clinically relevant 1 h timeframe. Furthermore, *ex vivo* studies with resected canine and human bladder tissues showed minimal signal from normal adjacent tissue and significant NIR fluorescence labeling of tumor tissue, in good agreement with our *in vitro* findings. Differential expression of EGFR *ex vivo* was revealed by our probe and confirmed by anti-EGFR immunohistochemical staining. Taken together, our data suggests Cy5.5-ELP-EGF is a NIR probe with improved sensitivity and selectivity towards BC that shows excellent potential for clinical translation.

## INTRODUCTION

Bladder cancer (BC) is the 10th most common malignancy, affecting more than half a million people worldwide each year, and accounts for 4.6% of the total new cancer cases in the United States [1]. With urothelial carcinoma (UC), the most common form of BC, the 5-year BC recurrence rate is nearly 78%, necessitating life-long surveillance, making it one of the costliest cancers to treat and manage [2, 3]. Cystoscopic imaging is used in the non-muscle invasive and muscle invasive forms of UC for initial detection and sampling, transurethral resection, and monitoring for recurrence following bladder sparing therapies. White light cystoscopy has low sensitivity (62–84%) and specificity (43–98%), contributing to a substantial percentage of false-negative findings [4, 5]. Hexaminolevulinate-based blue-light cystoscopy (BLC) allows better detection of urothelial carcinoma, however, it is associated with higher false-positive rates (6.1–39.3%), and in some cases, adverse events [4]. Bladder inflammation associated with chemotherapy, immunotherapy, or maintenance intravesical therapy can cause false positive findings with BLC [5, 6]. Bladder imaging research has been limited by the lack of appropriate pre-clinical animal models, especially larger animal models with naturally occurring heterogeneous tumors that mimic urothelial carcinoma in humans, that are suitable for cystoscopy [7, 8]. Dogs with naturally-occuring UC are an emerging option for a suitable large animal model of BC, where the cancer displays similar microscopic anatomy, histological appearance, biological behavior, heterogeneity, and molecular subtypes and markers to human invasive BC. Successful findings from research in dogs can be translated to humans, as well as being beneficial to pet dogs, leading to improved management of BC across the species [9, 10]. To facilitate advancement of successful results from the *in vitro* and *ex vivo* work presented here to the relevant *in vivo* canine model, experiments were performed in human and canine tissues and cells.

Overexpressed biomarkers in tumor tissue have been widely used for increasing the selectivity and sensitivity of tumor detection. One such biomarker in UC is epidermal growth factor receptor (EGFR), a rapidly internalizing receptor when bound to its ligand. EGFR is overexpressed in both human and canine urothelial carcinoma in about 75% of the cases [11, 12]. Thus, EGFR is a target for detection and treatment in non-muscle invasive and muscle invasive BC [13]. Near-infrared (NIR)-tagged antibodies against EGFR have shown promise by improving tumor-to-background signal compared to existing methods, but this approach suffers from production and purification issues as well as the need for creating species-specific analogs [10, 14–16]. Polypeptide-based probes have many favorable characteristics, such as higher tissue penetration, lower cost, tunable physiochemical properties, good target selectivity, and excellent biocompatibility [17–19]. Elastin-like polypeptides (ELP) are a promising class of materials that are finding increased biomedical applications due to their biocompatible, biodegradable, and low immunogenicity properties [20, 21]. Thompson and coworkers have previously reported a rapid purification method for ELP and ELP fusion proteins that yields pure material in a cost-efficient and scalable manner [22, 23].

**Scheme 1:**
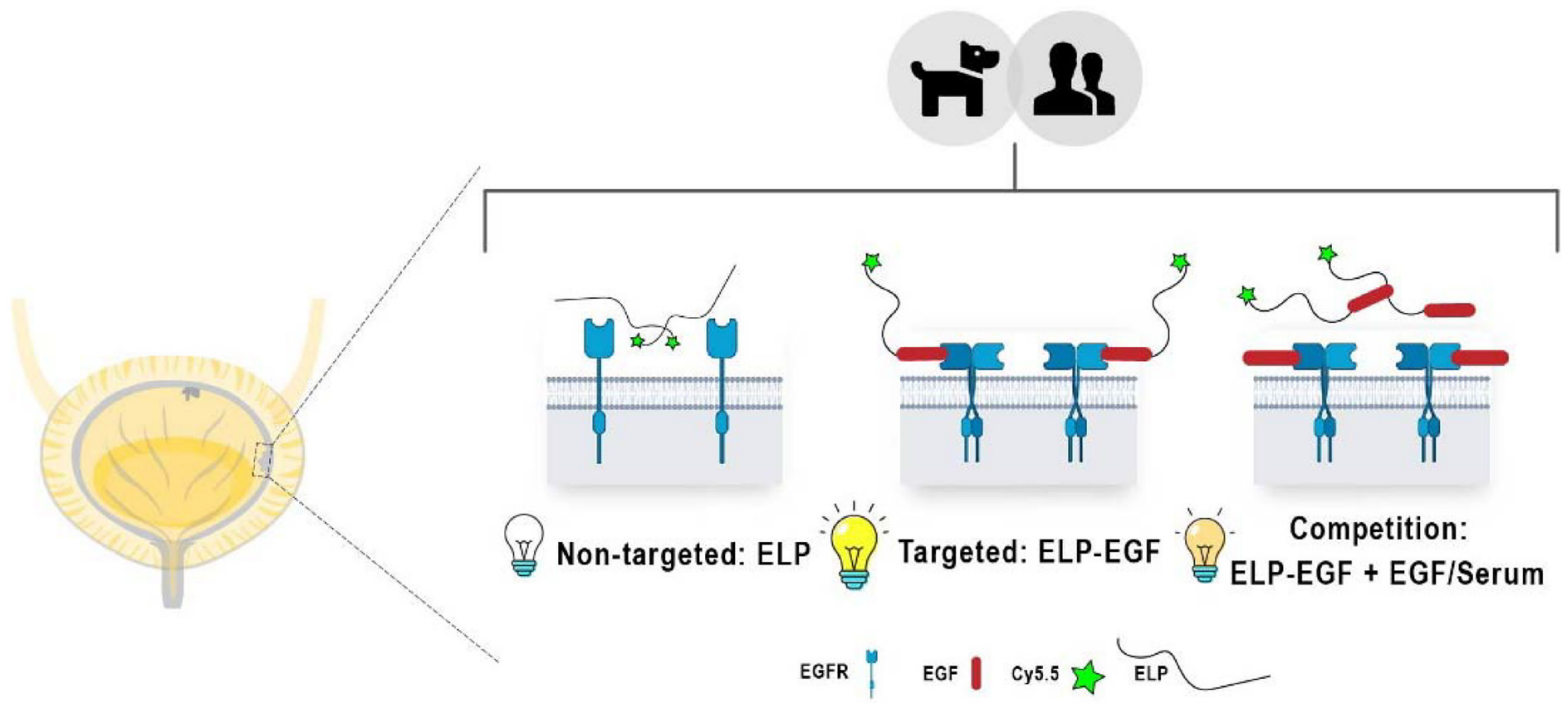
Local NIR emission expected (depicted as light bulbs of different intensities) for canine and human EGFR+ bladder cancer cells and tissues treated with non-targeted ELP (left), targeted ELP-EGF (center), and diminished ELP-EGF binding due to EGFR blockade with free EGF (right).

Here, we report the generation and performance of a NIR-tagged ELP-epidermal growth factor (ELP-EGF) fusion protein to target overexpressed EGFR in urothelial carcinoma (also known as transitional cell carcinoma, TCC) [12]. Our central hypothesis was that the ELP-EGF construct would bind to EGFR^+^ tumor cells with high affinity to enable NIR detection (Scheme 1). The selectivity of this targeting arises from two factors: overexpressed EGFR on the surface of the tumor cells and greater tumor access due to extensive mucosal barrier disruption at the bladder tumor site compared to normal urothelium. We show EGFR-specific binding and internalization in human and canine UC cell lines in EGFR density-dependent manner. These findings guided our experiments to evaluate *ex vivo* binding with intact spontaneous human and canine bladder tumor tissues and homogenates. *Ex vivo* findings followed the trends observed in our *in vitro* assays and were corroborated with the immunohistochemical anti-EGFR staining intensities of tissue slices. Furthermore, the whole tissue imaging results indicated that the non-targeted probe (Cy5.5-ELP) had no significant non-specific binding, while our EGFR-targeted probe displayed robust and specific EGFR binding.

## RESULTS

### Characterization of Cy5.5-N24-EGF and Cy5.5-N40

**Figure 1:**
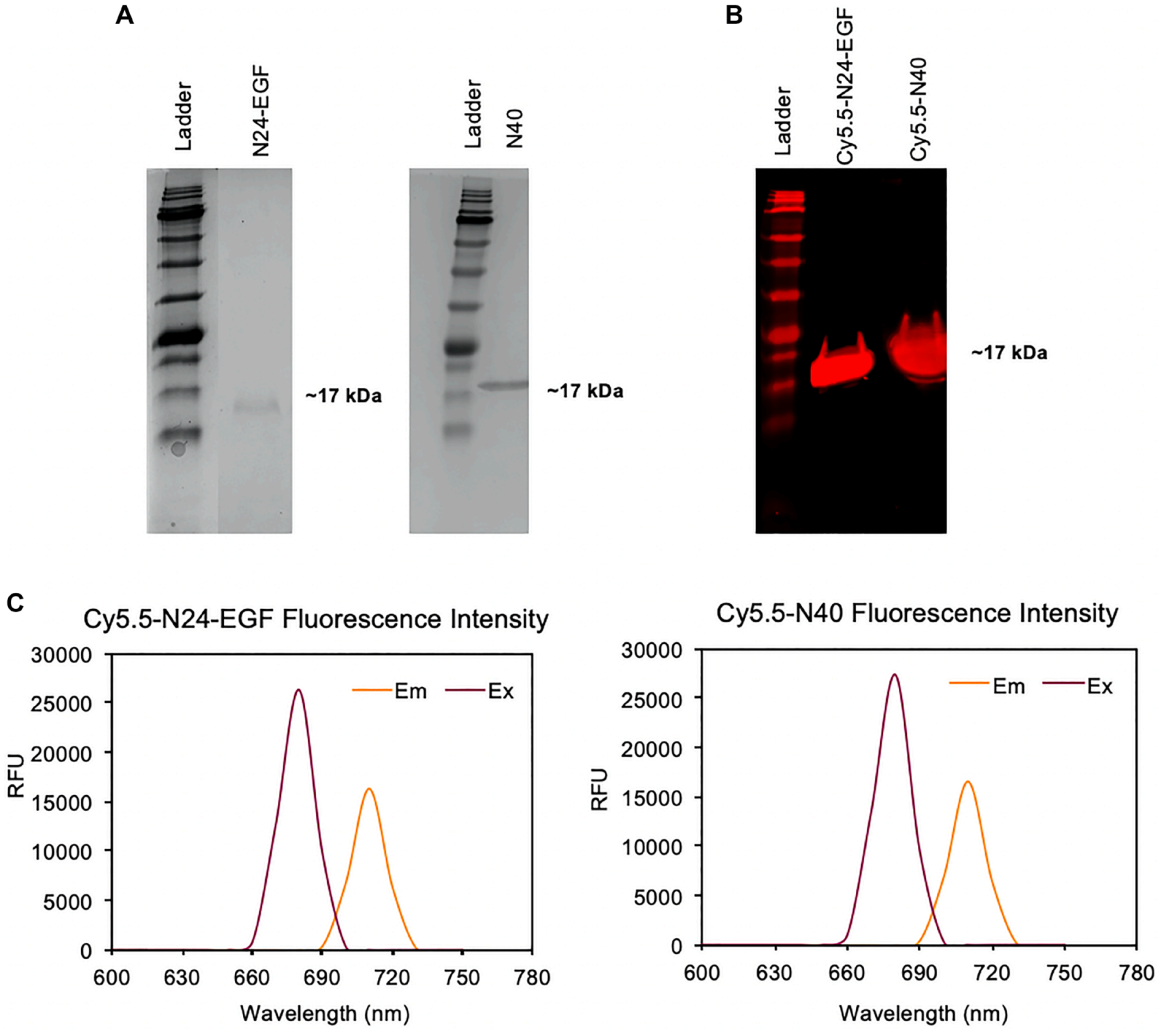
Characterization of Cy5.5-N24-EGF and Cy5.5-N40. (**A**) SDS-PAGE gels showing Cy5.5-N24-EGF and Cy5.5-N40 at ~17 kDa; (**B**) NIR fluorescence of SDS-PAGE gel after modification of N24-EGF and N40 with Cy5.5-NHS (λem- 700 nm); (**C**) Fluorescence excitation and emission spectra of purified Cy5.5-N24-EGF and Cy5.5-N40 (free Cy5.5: λex- 680 nm, λem- 710 nm).

Two ELP constructs of similar molecular weights, a targeted N24-EGF fusion and a non-targeted negative control N40 (Figure 1A) were purified as previously described [23] before chemically modifying them at their N-termini with Cy5.5 NHS ester to obtain Cy5.5-N24-EGF and Cy5.5-N40. SDS-PAGE analysis after purification by LH-20 Sephadex column chromatography revealed a single band of ~17 kDa under the NIR channel of a LICOR imaging system, confirming that both peptides were successfully modified (Figure 1B). Excitation and emission spectra were also recorded to determine the maximum excitation (680 nm) and emission (710 nm) wavelengths of the modified peptides; these values were used for all subsequent experiments (Figure 1C).

### *In vitro* binding studies with canine bladder cancer cell lines: K9TCC-SH (high EGFR) and K9TCC-Original (low EGFR)

**Figure 2:**
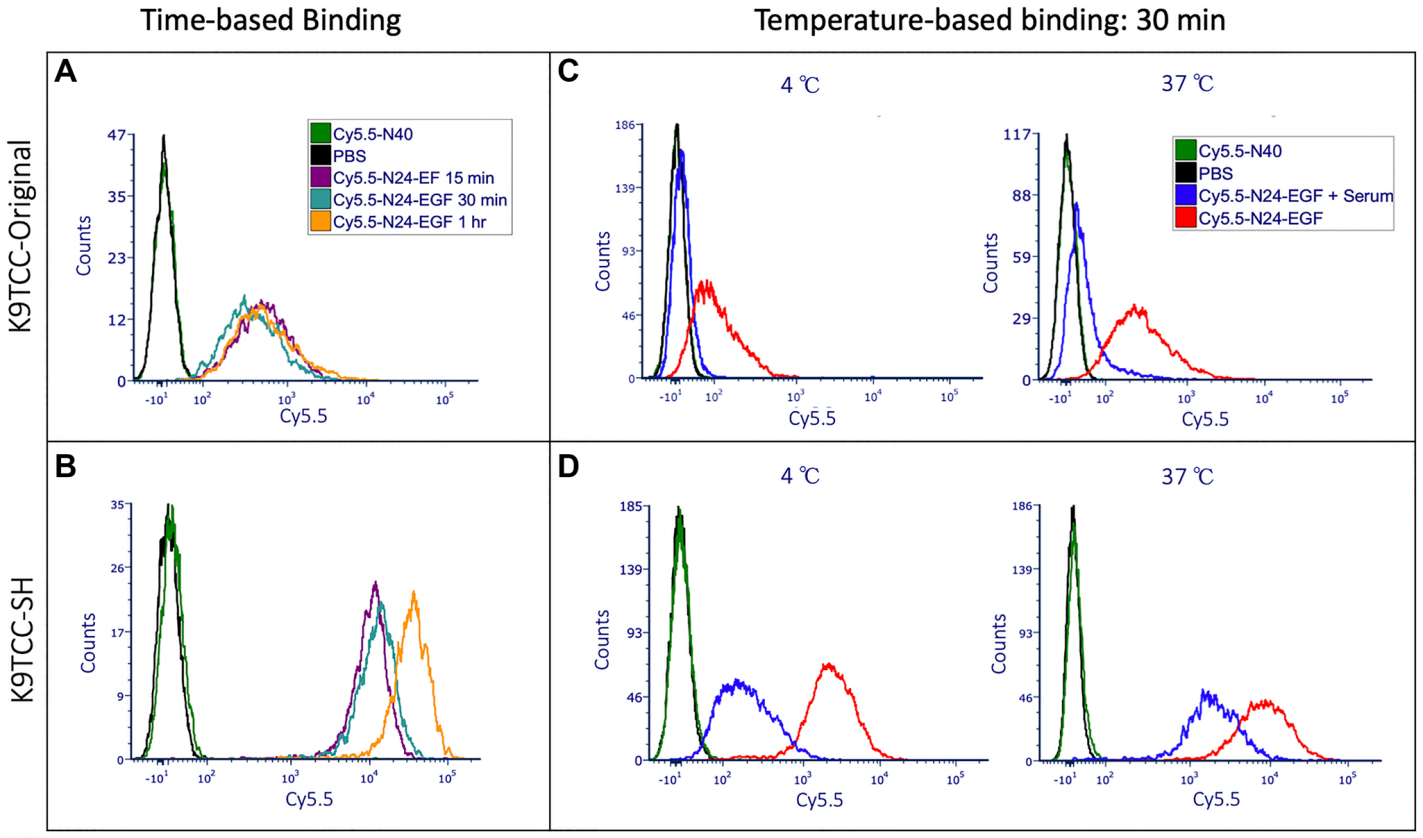
Time and temperature based *in vitro* binding studies of ELP constructs with K9TCC-Original (low EGFR) and K9TCC-SH (high EGFR). Here, PBS treated wells were used to adjust the voltages and gating in flow cytometry; Cy5.5-N40 is the negative control (i.e., similar molecular weight as Cy5.5-N24-EGF but without the EGF), and Cy5.5-N24-EGF is the ELP-EGF fusion protein. Competition experiments were performed by adding serum to the culture well along with Cy5.5-N24-EGF to evaluate whether receptor-mediated binding occurs. (**A**, **B**) Time-based (15 min, 30 min, 1 h) binding of K9TCC-Original and K9TCC-SH respectively; (**C**, **D**) Temperature-based (4°C and 37°C) binding of K9TCC-Original and K9TCC-SH, respectively.

Cell binding studies were performed with either Cy5.5-N24-EGF, Cy5.5-N40 or PBS by treating canine cell lines isolated from spontaneous canine bladder tumors that expressed low (K9TCC-Original) and high EGFR (K9TCC-SH), respectively [10]. An initial time-based binding study was performed over a 1 – 4 h period (Supplementary Figure 1). Saturation of the Cy5.5-N24-EGF fluorescence intensity was observed after 1 h. We also found that non-specific binding of Cy5.5-N40 increased after 1 h suggesting that experiments conducted using ≤ 1 h incubation with the Cy5.5-modified probes would be most reflective of EGFR^+^ cell binding. Next, we performed a binding study at 15, 30 and 60 mins to determine the probe binding kinetics. As shown in Figure 2A, Cy5.5-N24-EGF engages both canine cell lines within 15 min, with the K9TCC-SH cells displaying 5-fold higher fluorescence at 1 h than K9TCC-Original (Figure 2B and Supplementary Figure 2). In contrast, Cy5.5-N40 showed little to no binding after 1 h incubation with either cell line.

As a test for EGFR binding specificity by ELP-EGF, free EGF was added in a set of competition experiments. We found decreased binding of Cy5.5-N24-EGF when the culture media was supplemented with external EGF (Conc. = 200 μg/ml) at 30 min for K9TCC-SH and 15 min for K9TCC-Original (Supplementary Figure 2). Competition after 30 min was also observed in K9TCC-SH when serum was used instead of EGF as previously reported [10]. The difference in the EGFR expression by the two canine cell lines is reflected by the magnitude of Cy5.5-N24-EGF binding, showing about a 10-fold higher binding of Cy5.5-N24-EGF to K9TCC-SH compared to K9TCC-Original. Competition was also performed at 4°C to limit rapid (~5 min) internalization of the EGF-EGFR complex [24, 25]. Figure 2C, 2D show the results from competition studies of Cy5.5-N24-EGF at 4°C vs. 37°C for both cell lines. We observed that the lower temperature enhanced the competition with serum in K9TCC-SH (Figure 2D), however, minor changes were detected in the case of K9TCC-Original (Figure 2C). These binding patterns were sustained at other time points and temperatures studied as well (Supplementary Figures 3, 4).

### Confocal microscopy analysis of Cy5.5-N24-EGF internalization in K9TCC-SH and K9TCC-Original

**Figure 3:**
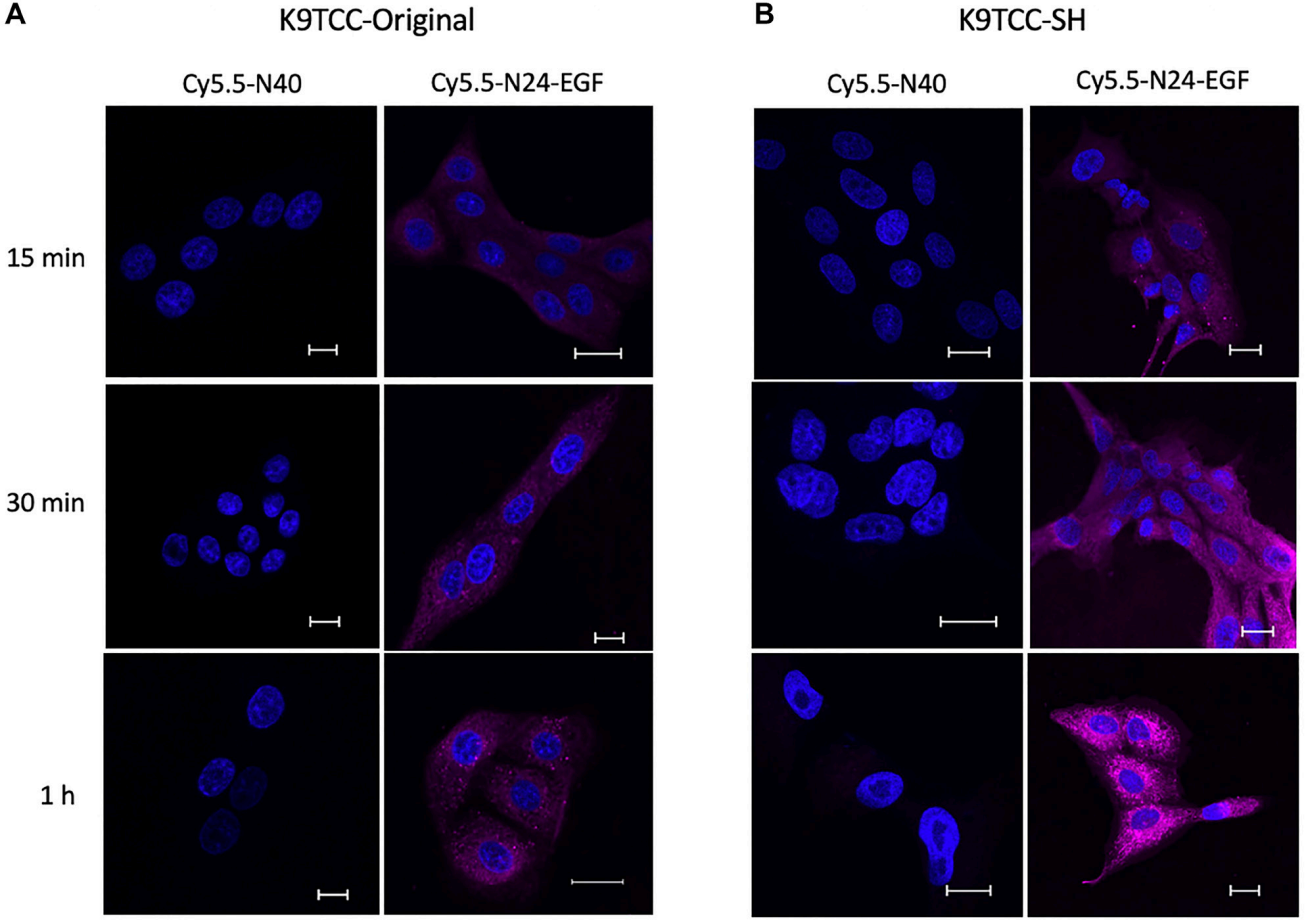
Confocal microscopy of internalized ELP constructs. Merged DAPI (blue) and NIR (fuchsia) confocal images for Cy5.5-N40 and Cy5.5-N24-EGF after incubating the cells for different times (15 min, 30 min, and 1 h) at the same probe concentration (180 μg/ml compound per 50k cells). Cy5.5-N24-EGF internalization increases with time for (**A**) K9TCC-Original and (**B**) K9TCC-SH. Scale bar: 20 μm. (Note: Confocal images for individual and merged channels appear in Supplementary Figures 5, 6)

After incubating the cells with either PBS, Cy5.5-N40, Cy5.5-N24-EGF, or Cy5.5-N24-EGF + Serum for different time periods at 37°C, they were analyzed for DAPI and Cy5.5 fluorescence by confocal microscopy (Figure 3, Supplementary Figures 5, 6). It was observed for both cell lines that the fluorescence intensities increased with increasing incubation time. Localization analysis revealed that the EGFR-targeted compound is bound and internalized within 15 mins, with longer incubation times leading to greater internalization. Cy5.5-N24-EGF internalization was reduced upon addition of serum at all time points recorded. The negative control, Cy5.5-N40, did not show any detectable internalization over 1 h.

### *In vitro* cell associated fluorescence studies with T24 human bladder cancer cells

#### Flow cytometry studies

**Figure 4:**
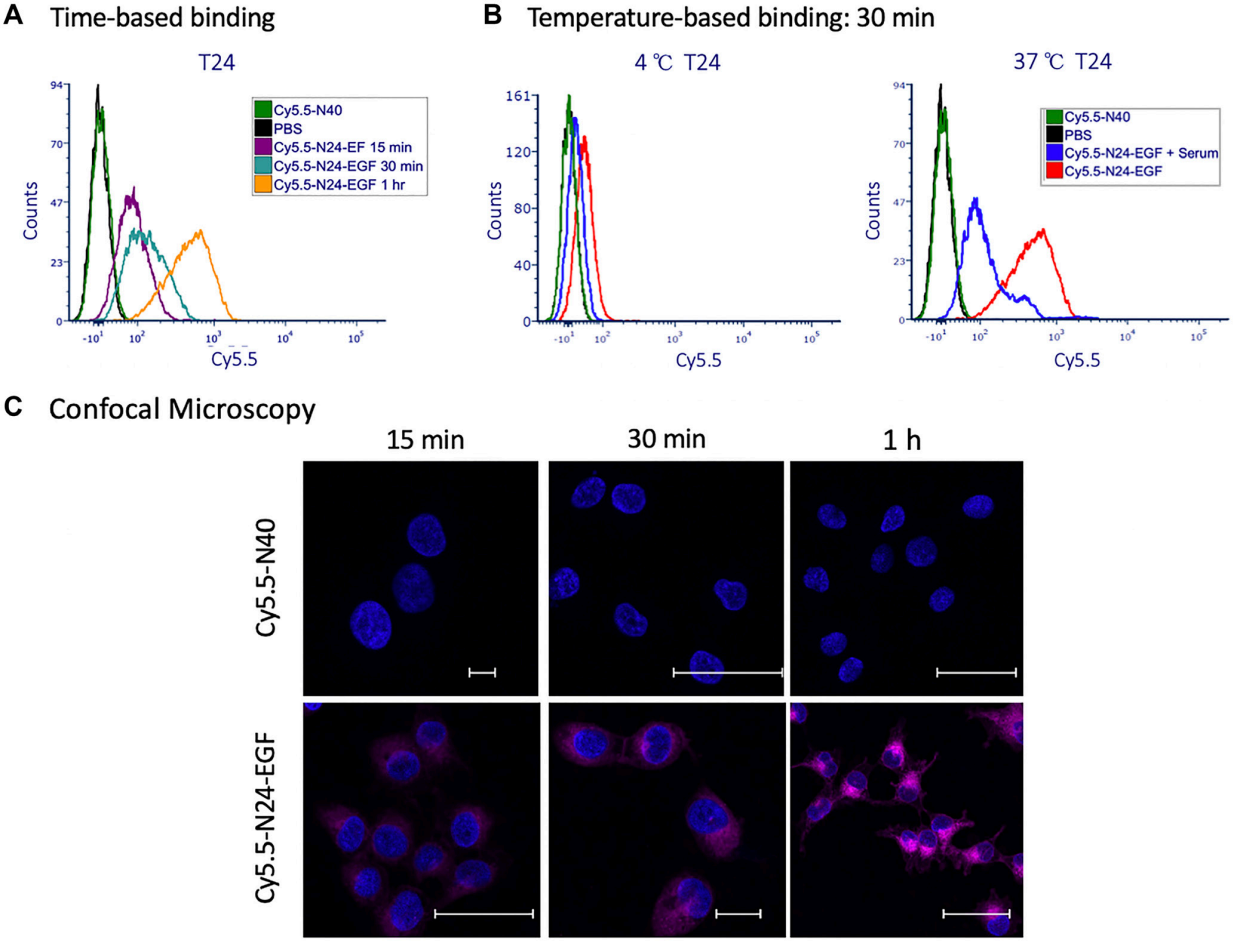
Binding of ELP probes to T24 cells as a function of time and temperature. (**A**) Time dependence of cell associated fluorescence after 15 min, 30 min and 1 h binding incubations with T24 cells at 37°C; (**B**) Temperature dependence of T24 cells associated fluorescence at 4°C and 37°C; (**C**) Merged DAPI (blue) and NIR (fuchsia) confocal images of T24 cells after incubation at 37°C with Cy5.5-N40 and Cy5.5-N24-EGF for different times (15 min, 30 min, 1 h). Scale bar: 20 μm. (Note: Confocal images for individual and merged channels appear in Supplementary Figure 7).

Cell-associated fluorescence of T24 cells incubated with Cy5.5-N24-EGF after incubation at 15, 30 and 60 min is shown in Figure 4A. At 37°C, Cy5.5-N24-EGF binds to T24 cells at levels that are similar to K9TCC-Original, the low EGFR expressing canine TCC cell line. Like the canine cells, blockade studies with Cy5.5-N24-EGF + serum produced lower mean fluorescence intensity compared to Cy5.5-N24-EGF, suggesting specific EGFR-mediated binding. Similar to our observations in canine cells, increased cell-associated Cy5.5 fluorescence was observed at higher incubation temperatures (Figure 4B). In contrast, T24 cells treated with Cy5.5-N40 (negative control) produced low mean fluorescence intensity (Figure 4C).

#### Confocal microscopy

T24 cells were treated with either PBS, Cy5.5-N40, Cy5.5-N24-EGF or Cy5.5-N24-EGF + Serum and evaluated by confocal microscopy for evidence of internalization. As observed in canine cell lines, internalization of Cy5.5-N24-EGF increases with time (Figure 4C, Supplementary Figure 7), whereas serum competition reduces the extent of Cy5.5-N24-EGF internalization. No detectable internalization was observed for Cy5.5-N40.

### *Ex vivo* binding studies with resected tumor and adjacent tissues

#### Canine tissue

**Figure 5:**
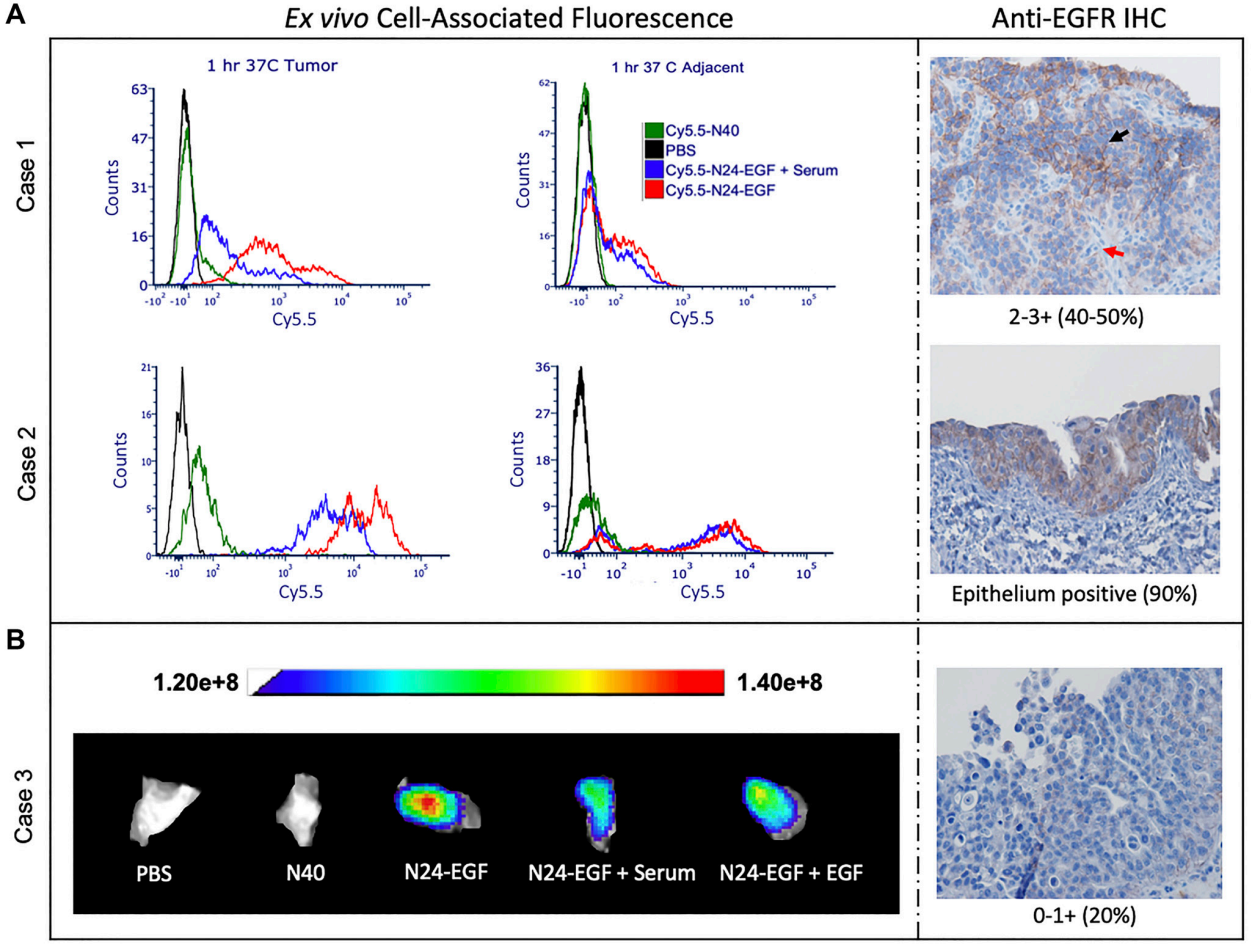
*Ex vivo* whole tissue imaging of canine bladder tumor tissue. (**A**) Homogenized tissue: two populations with varying intensities were observed for the Cy5.5-N24-EGF binding and corroborated by anti-EGFR IHC (Red arrow: Low EGFR; Black arrow: High EGFR). (**B**) Whole tissue: the tissue was divided into 5 pieces and incubated with different conditions (PBS, Cy5.5-N40, Cy5.5-N24-EGF, Cy5.5-N24-EGF + Serum, Cy5.5-N24-EGF + EGF) for 30 min. Fluorescence was recorded at λex: 640 nm and λem: 710 nm. Cy5.5-N24-EGF binding corroborates with anti-EGFR IHC.

Resected tumor and adjacent tissues were homogenized to produce single cell suspensions for flow cytometry analysis. Data were collected at three time points and two temperatures for tumor tissues whenever possible (Supplementary Figure 8). All samples were treated with either PBS, Cy5.5-N40, Cy5.5-N24-EGF or Cy5.5-N24-EGF + Serum. Cy5.5-N24-EGF showed the highest fluorescence, followed by Cy5.5-N24-EGF + Serum and Cy5.5-N40. The latter had little to no signal in all the cases, an observation that corroborated with the trend observed *in vitro* (Cases 1 and 2). Furthermore, in Case 1, two populations were observed at different mean fluorescence intensities for Cy5.5-N24-EGF binding (Figure 5A). This was validated by anti-EGFR staining that also showed two different EGFR+ populations. Whole tissue imaging was performed on 2 × 5 mm tissue sections after incubation with treatments mentioned above (Figure 5B). Each treatment group displayed similar Cy5.5 staining behavior as the homogenized tissue samples (Case 3). Anti-EGFR staining studies further confirmed the presence of EGFR+ cells.

#### Human tissue

**Figure 6:**
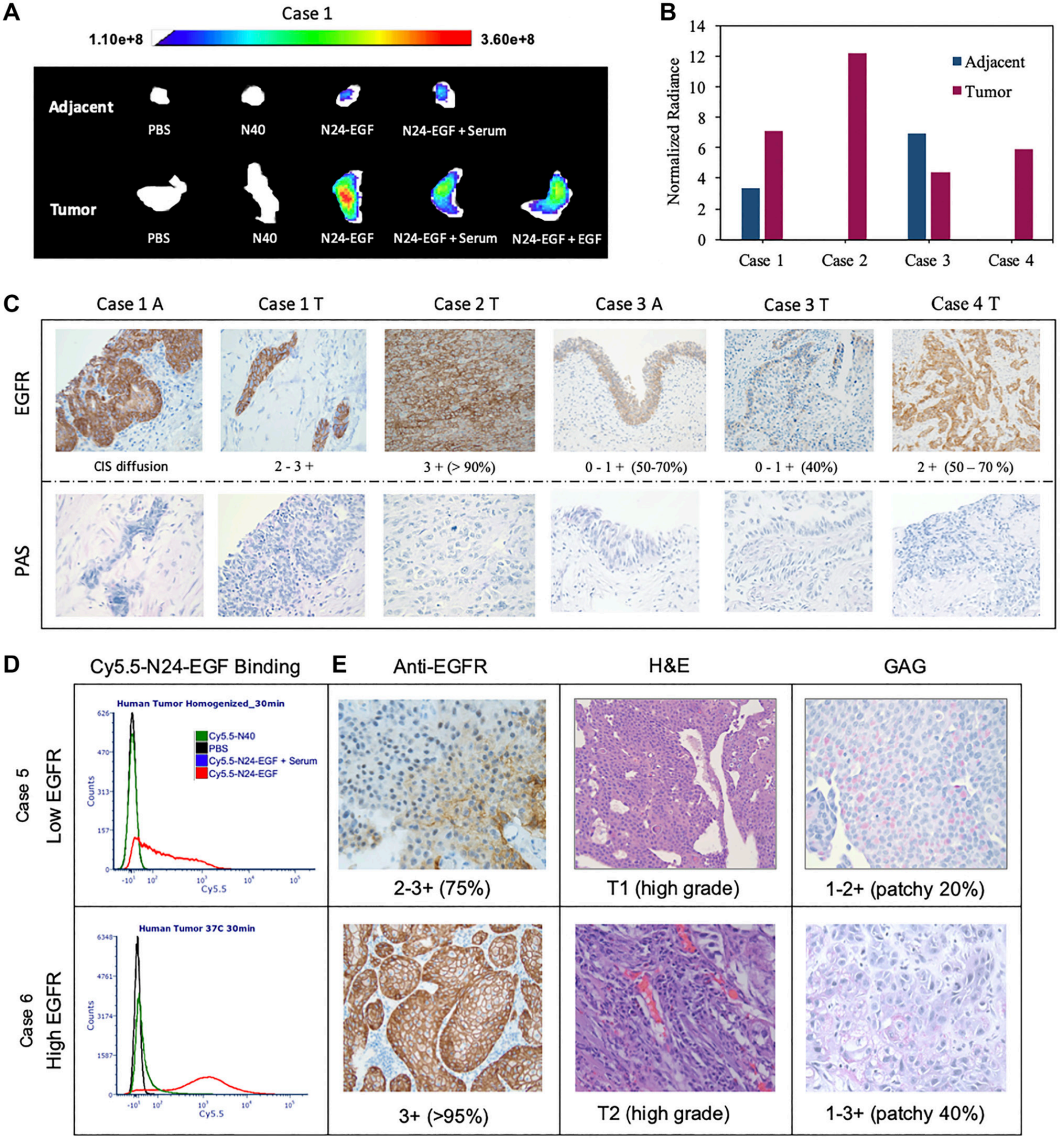
*Ex vivo* analysis of whole and homogenized human bladder tumor and adjacent tissue. (**A**) The tissues were divided into 5 pieces that were incubated under different probe conditions for 30 min. Cy5.5 fluorescence was recorded at λex: 640 nm and λem: 710 nm. (**B**) Mean fluorescence intensities of tissues treated with Cy5.5-N24-EGF after normalization against Cy5.5-N40 from 4 cases. Mean fluorescence intensities are reported as total fluorescence intensity within a region of interest normalized by tumor area. (**C**) Immunohistochemical analysis for EGFR and PAS (for GAG detection), with scoring of tissue sections by an independent core facility (T = tumor and A = Adjacent). (**D**) Homogenized tissue analysis: Two cases with different binding medians are shown here along (**E**) Images of Immunohistochemistry (IHC) for anti-EGFR, PAS and H&E staining. Differential EGFR expression was confirmed using ant-EGFR. The staining scores are written below the image and the numbers in bracket represent percentage of tumor cells that stained positive. [CIS- carcinoma *in situ*].

To assess the selectivity of the NIR probe for binding to tissue bearing EGFR^+^ cells, we compared their binding properties with human bladder tumor and adjacent tissues (Figure 6A, 6B, Supplementary Figure 9). Whole tissue imaging studies revealed that adjacent tissue displayed a lower average fluorescence intensity than tumor tissue when incubated for 30 min with Cy5.5-N24-EGF in all cases (Figure 6A, 6B). Furthermore, non-targeted Cy5.5-N40 did not produce significant binding over the same incubation period. Blockade studies performed to test receptor specificity indicated a reduction in mean fluorescence intensity of Cy5.5-N24-EGF in the presence of serum or external EGF. As a test of Cy5.5-ELP-EGF binding specificity, immunohistochemistry (IHC) was also performed to analyze EGFR expression and the presence/absence of glycosaminoglycan (GAG; Figure 6C). Anti-EGFR expression of adjacent tissue in Case 1 indicated diffused *carcinoma in-situ* and the anti-EGFR IHC scoring was 2–3+, findings that were consistent with our whole tissue imaging results. Comparisons between the cases (1–4) revealed that Case 2 had the highest binding and corresponding IHC scoring (3+; > 95% population), whereas Case 3 showed the lowest binding and score (0–1; > 40% population). In contrast, Case 4 had similar binding and IHC scoring (2+; 50–70%) as Case 1. Interestingly, Case 3 adjacent tissue had higher Cy5.5-N24-EGF signal compared to tumor, findings that were corroborated by anti-EGFR IHC scorings. All the samples showed sparse distribution of GAG via PAS staining. Similar trends were observed for homogenized tumors (Figure 6D, 6E, Supplementary Figure 9). Case 5 had lower EGFR compared to Case 6, an observation that was also reflected in the median fluorescence intensity of Cy5.5-N24-EGF binding in single cell analysis. Further, PAS staining indicated the presence of GAG in patches for both cases.

## DISCUSSION AND CONCLUSION

We have developed a peptide-based NIR probe, Cy5.5-N24-EGF, that shows high EGFR-specific binding specificity in canine and human UC *in vitro* and *ex vivo*. In all cases, the non-targeted ELP construct, Cy5.5-N40, showed minimal to no binding to tumor cells over a 60-min exposure, the saturation point for binding of the targeted probe. Cy5.5-N24-EGF showed a 100-10,000-fold fluorescence enhancement over Cy5.5-N40 in canine cell lines. This was a significant improvement compared to the 10-fold enhancement reported for an EGFR-antibody conjugated NIR agent in canine UC cell lines [10]. Some of the other approaches leveraging the EGF-EGFR axis, like EGF-toxin conjugates, have displayed a 10–50-fold enhancement [24, 25]. Competition studies were performed both *in vitro* and *ex vivo* at different temperatures in the presence of unlabeled serum and/or EGF. Temperature is known to influence the surface expression of EGFR and its cellular trafficking. At 4°C, in absence of growth factors, 90–95% of the expressed EGFR is found on the cell surface [26]. Furthermore, EGFR internalization is nearly abrogated at that temperature, whereas at 37°C, it falls in the range of 0.15–0.40 min^−1^ [27]. This high EGFR internalization rate necessitates EGF based competition at 4°C, especially for incubation times longer than 5 min [28]. While competition assays were performed at both temperatures, better competition was observed at 4°C in most cases.

At 37°C, Cy5.5-N24-EGF binding with human UC (T24) was very similar to the lower EGFR expressing canine UC (K9TCC-Original), whereas at 4°C, binding was lower in T24 compared to K9TCC-Original (Figures 2, 4), a finding that is consistent with previous studies showing low surface expression of EGFR in T24 at lower temperatures [29].

Our *ex vivo* binding results corroborate with the *in vitro* trends, regardless of whether we processed the resected tissues by enzymatic digestion or by direct treatment of the intact tissue (Figures 5, 6). In general, Cy5.5-N24-EGF showed lower binding to adjacent tissue specimens than their tumor counterparts. Furthermore, Cy5.5-N40 had no observable binding with any of the specimens over 1 h incubation period, a finding that we attribute to EGFR mediated interaction of the targeted probe. Additional support for an EGFR-specific interaction is provided by comparisons of the spatial fluorescence profiles of Cy5.5-N24-EGF and anti-EGFR binding on the same piece of tumor tissue. Tissue specimens were incubated with different treatments for 30 min, followed by acidic buffer washes to remove surface bound Cy5.5-N24-EGF before incubating the samples with anti-EGFR (Supplementary Figure 10) [30, 31]. We observed an overlap in the fluorescence profiles for Cy5.5-N24-EGF and anti-EGFR. Additional evidence to the binding outcomes of the probe came from IHC performed on the *ex vivo* specimens. The fluorescence binding intensities of our probe accounts for the differential expression of EGFR ranging from as low as 0–1 to as high as 3+ for human cases. Interestingly, we observed our probe stained high EGFR expressing (0–1+; 50–70% positive), T2a stage tissue specimen (Case 3A), marked as ‘adjacent’ during the conventional cystoscopy procedure.

In summary, we sought the development of Cy5.5-N24-EGF as an imaging probe for potential application during cystoscopy of bladder tumor by engaging overexpressed EGFR in tumor cells for various pre-clinical models. The probe achieves its maximum cell-associated fluorescence at 60 mins, a clinically relevant time frame for bladder instillation [32]. Owing to the high amino acid sequence homology (91%) between canine and human EGFR, no changes were required in the ELP sequence for translation across species [33]. Our results provide empirical support for EGFR-mediated interaction of the probe based on: (*i*) Cy5.5-N24-EGF produced high cell-associated fluorescence, (*ii*) Cy5.5-N24-EGF could be competed away from EGFR+ cells with exogenous EGF, and (*iii*) positive correlation was observed between EGFR levels from IHC and Cy5.5-N24-EGF binding intensities in human and canine *ex vivo* tissues. EGFR targeting approaches are effective in 74% of bladder cancer patients [34–36]. While this initial proof-of-concept work focused on EGFR, future work will expand this approach by developing ELP based multi-ligand probes targeting more tumor markers. This will facilitate imaging in patients with a variety of molecular targets. By demonstrating the binding efficacy in canine tissue and cells this will set the stage for imaging canine tumors during cystoscopy in the future and enable translation to humans.

## MATERIALS AND METHODS

### Regulatory approvals

Biospecimen Collection and Banking Core at the IU Simon Comprehensive Cancer Center provided human urothelial tissue samples service in support of this study. All canine tissue samples were obtained following the guidelines and approval of the Purdue Animal Care and Use Committee. Tissue samples were collected at necropsy from dogs who were euthanized at the request of their owner due to poor quality of life from cancer or other comorbidities.

### Chemicals and reagents

All organic solvents (methanol, ethanol, acetone, dimethyl formamide (DMF) and, N,N-diisopropylethylamine (DIPEA)) were HPLC grade (Fisher Scientific) and used without further purification. *E. coli* cells (BL21 and NEB 5-alpha) were purchased from New England Biolabs. Lysozyme and Sephadex LH-20 were purchased from Sigma-Aldrich. BLUEStain protein ladder and ampicillin were purchased from Gold Biotechnology. Yeast extract, L-proline, tryptone, glycerol, and other bacterial culture media components were purchased from Research Products International. All materials for SDS-PAGE were obtained from Bio-Rad. PE/Cyanine7 anti-human EGFR antibody was purchased from BioLegend. Cy5.5 NHS ester was obtained from Lumi Probe. DMEM/F12, DMEM, fetal bovine serum (FBS), PenStrep, and other cell culture reagents were obtained from Corning Life Sciences.

### Protein expression and purification

All protein expressions were performed using BL21(DE3) *E. coli* and cultured as previously reported [23]. N24-EGF was purified by organic extraction (Ethanol: Methanol, 1:5 ratio) following a previously reported protocol [23]. For N40 purification, conventional inverse transition cycling was performed as described [22].

### Fluorophore modification

Purified N24-EGF or N40 pellets were resuspended in DMF, followed by addition of 1 eq. DIPEA base and 2 eq. of Cy5.5 NHS ester to the resuspended ELP solution (1 g pellet in 200 μL DMF). The mixture was stirred overnight at 4°C. Excess Cy5.5 was removed using size exclusion chromatography (LH-20 Sephadex) as per the manufacturer’s recommendations. Fluorescence intensities (λ_ex_: 681 nm/λ_em_: 710 nm) of Cy5.5-N24-EGF and Cy5.5-N40 were normalized prior to cell treatments.

### SDS PAGE Analysis and NIR imaging

Proteins were electrophoresed through a 15% resolving gel with a 5% stacking gel in a Tris-glycine buffer system and stained following a previous protocol [23]. The gels were then imaged using a Bio-Rad Chemidoc Touch imaging system. For NIR imaging, the fractions post-workup were run on the gel as described above and imaged with a LICOR imaging system using the 700 nm emission channel.

### *In vitro* analysis

#### Cell culture

The canine transitional bladder cancer cell lines, K9TCC-Original (minimal EGFR expression) and K9TCC-SH (intermediate EGFR expression) were provided by the Knapp Lab (Purdue University, West Lafayette, IN, US) and grown as previously described [10]. The human urothelial carcinoma cell line, T24 (procured from ATCC), was kindly provided by Dr. Timothy Ratliff (Purdue University, West Lafayette, IN, US) and cultured in McCoy’s medium supplemented with 10% FBS at 37°C and 5% CO_2_. All experiments were performed with mycoplasma-free cells.

#### Binding assay

Canine cancer cells were seeded at a density of 50,000 cells/well in 24-well plates overnight in serum containing DMEM/F12 and allowed to attain 80% confluency. The wells were then rinsed 3 times with 1X PBS. Further, 100 μL of different conditions (PBS, Cy5.5-N40 and Cy5.5-N24-EGF) were diluted to 400 μL serum free media and then introduced to the cells. For serum competition studies, 100 μL of Cy5.5-N24-EGF was diluted in 400 μL of 20% serum containing media and added to the cells. Each condition was incubated for either 15, 30 or 60 mins at either 4°C or 37°C. For free EGF competition, a 2-fold excess of EGF relative to Cy5.5-N24-EGF was used and allowed to incubate for the desired time and temperature. Post incubation, the cells were rinsed 3X with PBS and trypsinized (100 μL per well) for 10 min at 37°C. FACS buffer (500 μL) was used to neutralize the trypsin and filtered through tubes with a cell strainer cap to minimize clumping. Samples were evaluated using a BD LSRFortessa cell analyzer using the APC-Alexa 700 channel (λ_ex_ = 640 nm/λ_em_ = 710 nm); the data was further processed using FCS Express 7.

#### Confocal microscopy

Poly-L-lysine coated slides were placed in a 6 well plate and 150 k cells/well were seeded and incubated in serum containing DMEM media until 80% confluency was reached. Time based internalization studies were performed for the same conditions and concentrations as the binding studies. All the coverslips were fixed with 2% paraformaldehyde for 15 min and washed with 1x PBS post-fixation before mounting on the slides using ibidi mounting media (DAPI staining). All the slides were analyzed with a Nikon AIR-MP microscope under the DAPI and Cy5.5 channels.

### *Ex vivo* analysis

#### Tissue- derived single analysis by flow cytometry

*Ex vivo* bladder tumor and adjacent tissue samples were obtained from humans or dogs with naturally occurring invasive UC. Human *ex vivo* samples were obtained from Indiana University Melvin and Bren Simon Comprehensive Cancer Center-Tissue Procurement and Distribution Core. The canine tissues (canine tissue: cases 1, 2) were collected at necropsy; human tissue was obtained (human tissue: cases 5, 6) by transurethral resection of bladder tumor. A single cell suspension was prepared using Accumax enzymatic digestion (30 min, room temp) and distributed into the following treatments: PBS, Cy5.5-N40, Cy5.5-N24-EGF, Cy5.5-N24-EGF + Serum/EGF. All the conditions were allowed to incubate for 15, 30 or 60 min at either 37 or 4°C. Post-incubation, cells were washed with PBS and centrifuged at 1500 rpm for 5 min. The cell pellet was re-suspended in 500 μL FACS buffer and passed through a cell strainer cap for flow cytometry analysis. To each flow tube, 5 μL of propidium iodide (10 μg/ml) was added for 5–10 min to stain the dead cell population. All the samples were evaluated using a BD LSRFortessa cell analyzer with the APC-Alexa 700 and PI (λ_ex_: 537 nm/λ_em_: 635 nm) channels and the data processed using FCS Express 7.

#### Whole tissue imaging

Whole tissues were procured from canine necropsy (Case 3) and human cystectomy (Case 1–4) patients. In some cases, adjacent tissues appearing normal were also collected. The samples were washed three times with PBS before sectioning (5 mm × 2 mm) and submerging in 300 μL serum free media along with 200 μL PBS, Cy5.5-N40, or Cy5.5-N24-EGF. After sample incubation and fixation, NIR images were collected with an Aura animal imager using the λ_ex_ = 680 nm/λ_em_ = 710 nm channel.

#### Immunohistochemistry studies

All the IHC for human samples were performed by the IU Immunohistochemistry Core facility. For Canine cases, the IHC was conducted in the Knapp lab and in the Indiana Animal Disease and Diagnostic Laboratory at Purdue University.

NOTE: A more detailed materials and methods section is available in supplementary information.

## SUPPLEMENTARY MATERIALS



## Abbreviations

NIR: near infrared
EGFR: epidermal growth factor receptor
UC: urothelial carcinoma
ELP: Elastin-like polypeptide
EGF: epidermal growth factor
DMF: dimethyl formamide
DIPEA: N,N-diisopropylethylamine
SDS-PAGE: sodium dodecyl sulfate-polyacrylamide gel electrophoresis
BLC: blue light cystoscopy
TCC: transitional cell carcinoma, IHC: Immunohistochemistry
N24-EGF: (VPGVG)24-Epidermal growth factor
N40: (VPGVG)40
H&E: hematoxylin and eosin
GAG: glycosaminoglycan
PAS: Periodic acid-Schiff stain

## References

[1] Saginala K , Barsouk A , Aluru JS , Rawla P , Padala SA , Barsouk A . Epidemiology of Bladder Cancer. Med Sci (Basel). 2020; 8:15. 10.3390/medsci8010015 32183076 PMC7151633

[2] Pan Y , Volkmer JP , Mach KE , Rouse RV , Liu JJ , Sahoo D , Chang TC , Metzner TJ , Kang L , van de Rijn M , Skinner EC , Gambhir SS , Weissman IL , Liao JC . Endoscopic molecular imaging of human bladder cancer using a CD47 antibody. Sci Transl Med. 2014; 6:260ra148. 10.1126/scitranslmed.3009457 25355698

[3] D’Andrea D , Soria F , Zehetmayer S , Gust KM , Korn S , Witjes JA , Shariat SF . Diagnostic accuracy, clinical utility and influence on decision-making of a methylation urine biomarker test in the surveillance of non-muscle-invasive bladder cancer. BJU Int. 2019; 123:959–67. 10.1111/bju.14673 30653818 PMC6850401

[4] Shining a Light on Blue Light Cystoscopy with Hexvix^®^/Cysview^®^: What You Need to Know. 2021. https://www.cysview.com/info-for-hcps/procedure-hints-and-tips/.

[5] Pietzak EJ . The Impact of blue light cystoscopy on the diagnosis and treatment of bladder cancer. Curr Urol Rep. 2017; 18:39. 10.1007/s11934-017-0685-8 28324275

[6] Jarow JP , Lerner SP , Kluetz PG , Liu K , Sridhara R , Bajorin D , Chang S , Dinney CP , Groshen S , Morton RA , O’Donnell M , Quale DZ , Schoenberg M , et al. Clinical trial design for the development of new therapies for nonmuscle-invasive bladder cancer: report of a Food and Drug Administration and American Urological Association public workshop. Urology. 2014; 83:262–64. 10.1016/j.urology.2013.10.030 24332121

[7] Cekanova M , Uddin MJ , Bartges JW , Callens A , Legendre AM , Rathore K , Wright L , Carter A , Marnett LJ . Molecular imaging of cyclooxygenase-2 in canine transitional cell carcinomas in vitro and in vivo. Cancer Prev Res (Phila). 2013; 6:466–76. 10.1158/1940-6207.CAPR-12-0358 23531445 PMC3671760

[8] Cekanova M , Rathore K . Animal models and therapeutic molecular targets of cancer: utility and limitations. Drug Des Devel Ther. 2014; 8:1911–21. 10.2147/DDDT.S49584 25342884 PMC4206199

[9] Knapp DW , Ramos-Vara JA , Moore GE , Dhawan D , Bonney PL , Young KE . Urinary bladder cancer in dogs, a naturally occurring model for cancer biology and drug development. ILAR J. 2014; 55:100–18. 10.1093/ilar/ilu018 24936033

[10] Nagaya T , Okuyama S , Ogata F , Maruoka Y , Knapp DW , Karagiannis SN , Fazekas-Singer J , Choyke PL , LeBlanc AK , Jensen-Jarolim E , Kobayashi H . Near infrared photoimmunotherapy targeting bladder cancer with a canine anti-epidermal growth factor receptor (EGFR) antibody. Oncotarget. 2018; 9:19026–38. 10.18632/oncotarget.24876 29721181 PMC5922375

[11] Hanazono K , Fukumoto S , Kawamura Y , Endo Y , Kadosawa T , Iwano H , Uchide T . Epidermal growth factor receptor expression in canine transitional cell carcinoma. J Vet Med Sci. 2015; 77:1–6. 10.1292/jvms.14-0032 25223345 PMC4349531

[12] Knapp DW , Dhawan D , Ramos-Vara JA , Ratliff TL , Cresswell GM , Utturkar S , Sommer BC , Fulkerson CM , Hahn NM . Naturally-occurring invasive urothelial carcinoma in dogs, a unique model to drive advances in managing muscle invasive bladder cancer in humans. Front Oncol. 2019; 9:1493. 10.3389/fonc.2019.01493 32039002 PMC6985458

[13] Railkar R , Krane LS , Li QQ , Sanford T , Siddiqui MR , Haines D , Vourganti S , Brancato SJ , Choyke PL , Kobayashi H , Agarwal PK . Epidermal growth factor receptor (egfr)-targeted photoimmunotherapy (PIT) for the treatment of egfr-expressing bladder cancer. Mol Cancer Ther. 2017; 16:2201–14. 10.1158/1535-7163.MCT-16-0924 28619755 PMC5628127

[14] Siddiqui MR , Railkar R , Sanford T , Crooks DR , Eckhaus MA , Haines D , Choyke PL , Kobayashi H , Agarwal PK . Targeting epidermal growth factor receptor (EGFR) and human epidermal growth factor receptor 2 (HER2) expressing bladder cancer using combination photoimmunotherapy (PIT). Sci Rep. 2019; 9:2084. 10.1038/s41598-019-38575-x 30765854 PMC6375935

[15] Lee YJ , Jeong KJ . Challenges to production of antibodies in bacteria and yeast. J Biosci Bioeng. 2015; 120:483–90. 10.1016/j.jbiosc.2015.03.009 25912450

[16] Singer J , Fazekas J , Wang W , Weichselbaumer M , Matz M , Mader A , Steinfellner W , Meitz S , Mechtcheriakova D , Sobanov Y , Willmann M , Stockner T , Spillner E , et al. Generation of a canine anti-EGFR (ErbB-1) antibody for passive immunotherapy in dog cancer patients. Mol Cancer Ther. 2014; 13:1777–90. 10.1158/1535-7163.MCT-13-0288 24755200 PMC4174294

[17] Chen H , Zhang S , Lv X , Guo S , Ma Y , Han B , Hu X . Interactions between suspended sediments and submerged macrophytes-epiphytic biofilms under water flow in shallow lakes. Water Res. 2022; 222:118911. 10.1016/j.watres.2022.118911 35932704

[18] Sato AK , Viswanathan M , Kent RB , Wood CR . Therapeutic peptides: technological advances driving peptides into development. Curr Opin Biotechnol. 2006; 17:638–42. 10.1016/j.copbio.2006.10.002 17049837

[19] Ducharme M , Lapi SE . Peptide based imaging agents for her2 imaging in oncology. Mol Imaging. 2020; 19:1536012120960258. 10.1177/1536012120960258 32957830 PMC7513396

[20] Mead BP , Mastorakos P , Suk JS , Klibanov AL , Hanes J , Price RJ . Targeted gene transfer to the brain via the delivery of brain-penetrating DNA nanoparticles with focused ultrasound. J Control Release. 2016; 223:109–17. 10.1016/j.jconrel.2015.12.034 26732553 PMC4739627

[21] Varanko AK , Su JC , Chilkoti A . Elastin-like polypeptides for biomedical applications. Annu Rev Biomed Eng. 2020; 22:343–69. 10.1146/annurev-bioeng-092419-061127 32343908

[22] VerHeul R , Sweet C , Thompson DH . Rapid and simple purification of elastin-like polypeptides directly from whole cells and cell lysates by organic solvent extraction. Biomater Sci. 2018; 6:863–76. 10.1039/c8bm00124c 29488993 PMC5869169

[23] Sweet C , Aayush A , Readnour L , Solomon KV , Thompson DH . Development of a fast organic extraction-precipitation method for improved purification of elastin-like polypeptides that is independent of sequence and molecular weight. Biomacromolecules. 2021; 22:1990–98. 10.1021/acs.biomac.1c00096 33826307 PMC8496954

[24] Masilamani AP , Fischer A , Schultze-Seemann S , Kuckuck I , Wolf I , Dressler FF , Gratzke C , Wolf P . Epidermal growth factor based targeted toxin for the treatment of bladder cancer. Anticancer Res. 2021; 41:3741–46. 10.21873/anticanres.15165 34281832

[25] Michalska M , Schultze-Seemann S , Bogatyreva L , Hauschke D , Wetterauer U , Wolf P . In vitro and in vivo effects of a recombinant anti-PSMA immunotoxin in combination with docetaxel against prostate cancer. Oncotarget. 2016; 7:22531–42. 10.18632/oncotarget.8001 26968813 PMC5008379

[26] Burke P , Schooler K , Wiley HS . Regulation of epidermal growth factor receptor signaling by endocytosis and intracellular trafficking. Mol Biol Cell. 2001; 12:1897–910. 10.1091/mbc.12.6.1897 11408594 PMC37350

[27] Moehren G , Markevich N , Demin O , Kiyatkin A , Goryanin I , Hoek JB , Kholodenko BN . Temperature dependence of the epidermal growth factor receptor signaling network can be accounted for by a kinetic model. Biochemistry. 2002; 41:306–20. 10.1021/bi011506c 11772030

[28] Sorkin A , Duex JE . Quantitative analysis of endocytosis and turnover of epidermal growth factor (EGF) and EGF receptor. Curr Protoc Cell Biol. 2010; Chapter 15:Unit 15.14. 10.1002/0471143030.cb1514s46 20235100 PMC2878126

[29] Qu XJ , Yang JL , Russell PJ , Goldstein D . Changes in epidermal growth factor receptor expression in human bladder cancer cell lines following interferon-alpha treatment. J Urol. 2004; 172:733–38. 10.1097/01.ju.0000130751.83953.55 15247772

[30] Honegger AM , Dull TJ , Felder S , Van Obberghen E , Bellot F , Szapary D , Schmidt A , Ullrich A , Schlessinger J . Point mutation at the ATP binding site of EGF receptor abolishes protein-tyrosine kinase activity and alters cellular routing. Cell. 1987; 51:199–209. 10.1016/0092-8674(87)90147-4 3499230

[31] Roepstorff K , Grandal MV , Henriksen L , Knudsen SL , Lerdrup M , Grøvdal L , Willumsen BM , van Deurs B . Differential effects of EGFR ligands on endocytic sorting of the receptor. Traffic. 2009; 10:1115–27. 10.1111/j.1600-0854.2009.00943.x 19531065 PMC2723868

[32] Grossman HB , Stenzl A , Fradet Y , Mynderse LA , Kriegmair M , Witjes JA , Soloway MS , Karl A , Burger M . Long-term decrease in bladder cancer recurrence with hexaminolevulinate enabled fluorescence cystoscopy. J Urol. 2012; 188:58–62. 10.1016/j.juro.2012.03.007 22583635 PMC3372634

[33] Singer J , Weichselbaumer M , Stockner T , Mechtcheriakova D , Sobanov Y , Bajna E , Wrba F , Horvat R , Thalhammer JG , Willmann M , Jensen-Jarolim E . Comparative oncology: ErbB-1 and ErbB-2 homologues in canine cancer are susceptible to cetuximab and trastuzumab targeting. Mol Immunol. 2012; 50:200–9. 10.1016/j.molimm.2012.01.002 22424313 PMC3318186

[34] Siddiqui MR , Railkar R , Sanford T , Crooks DR , Eckhaus MA , Haines D , Choyke PL , Kobayashi H , Agarwal PK . Targeting Epidermal Growth Factor Receptor (EGFR) and Human Epidermal Growth Factor Receptor 2 (HER2) Expressing Bladder Cancer Using Combination Photoimmunotherapy (PIT). Sci Rep. 2019; 9:2084. 10.1038/s41598-019-38575-x 30765854 PMC6375935

[35] Buss JH , Begnini KR , Bruinsmann FA , Ceolin T , Sonego MS , Pohlmann AR , Guterres SS , Collares T , Seixas FK . Lapatinib-loaded nanocapsules enhances antitumoral effect in human bladder cancer cell. Front Oncol. 2019; 9:203. 10.3389/fonc.2019.00203 31024833 PMC6465636

[36] Alessandrino F , Ghaith O , Williams K , Sonpavde GP , Silverman SG , Shinagare AB . Advanced urothelial cancer: a radiology update. Abdom Radiol (NY). 2019; 44:3858–73. 10.1007/s00261-019-02148-3 31363813

