# Supplementary material for: Targeted elastin-like polypeptide fusion protein for near-infrared imaging of human and canine urothelial carcinoma
Source: Oncotarget. 2022 Sep 6;13:1004–16. doi: 10.18632/oncotarget.28271 (PMC9447490; doi:10.18632/oncotarget.28271)
Supplement: Supplementary file 1 [file oncotarget-13-28271-s001.pdf]

Targeted elastin-like polypeptide fusion protein for near-infrared imaging of human and canine urothelial carcinoma

SUPPLEMENTARY MATERIALS

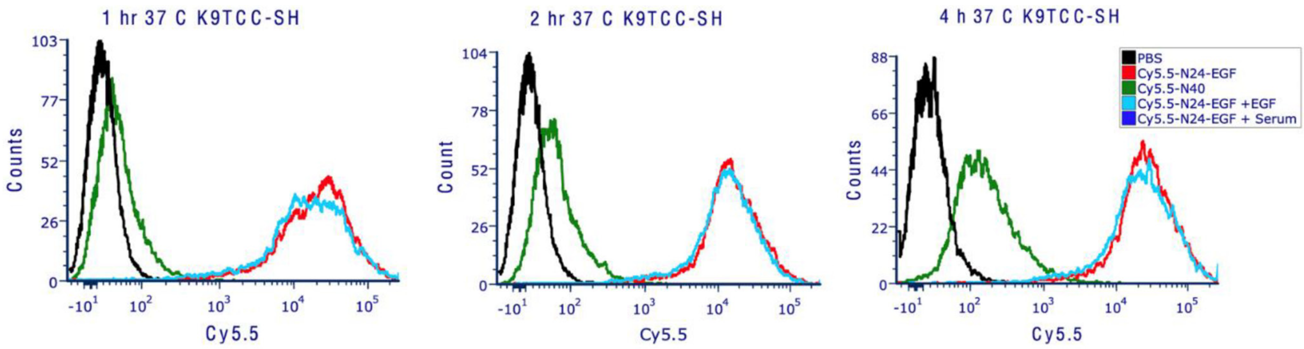

Supplementary Figure 1: Time based binding study performed with canine K9TCC-SH cells over 1 – 4 h at 37°C. The binding of Cy5.5-N24-EGF remained constant after 1 h, suggesting that the probe saturates within 1 h. Competition with free EGF showed little change in cell-associated fluorescence. Cy5.5-N40 binding increased after 1 h, with maximum binding at 4 h, suggesting that non-specific adsorption of this construct occurs at longer incubation times.

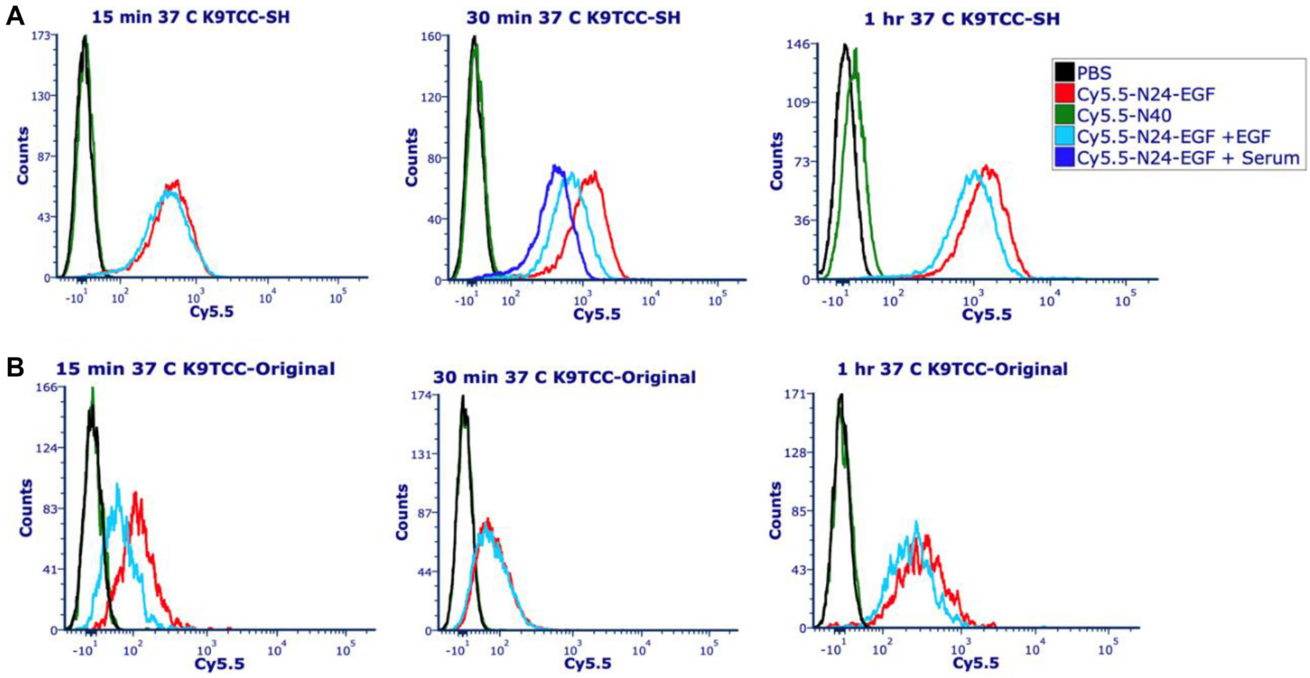

Supplementary Figure 2: Time based binding study performed in canine transitional carcinoma cells over 15, 30, and 60 min. (**A**) K9TCC-SH: Cy5.5-N24-EGF bound the cells within 15 min and reached saturation by 1 h. Competition studies at 30 min indicated that Cy5.5-N24-EGF + EGF had lower binding compared to Cy5.5-N24-EGF, whereas serum challenge had lower cell-associated fluorescence than Cy5.5-N24-EGF or Cy5.5-N24-EGF + EGF. Cy5.5-N40 was similar to PBS controls in all cases. (**B**) K9TCC-Original: In this low EGFR expressing cell line, Cy5.5-N24-EGF binding reached saturation by 1 h. Blockade studies indicated that receptor competition occurs within 15 min and Cy5.5-N40 binding was similar to PBS controls. Overall, Cy5.5-N24-EGF binding to K9TCC-SH was ~5-fold higher than to K9TCC-Original.

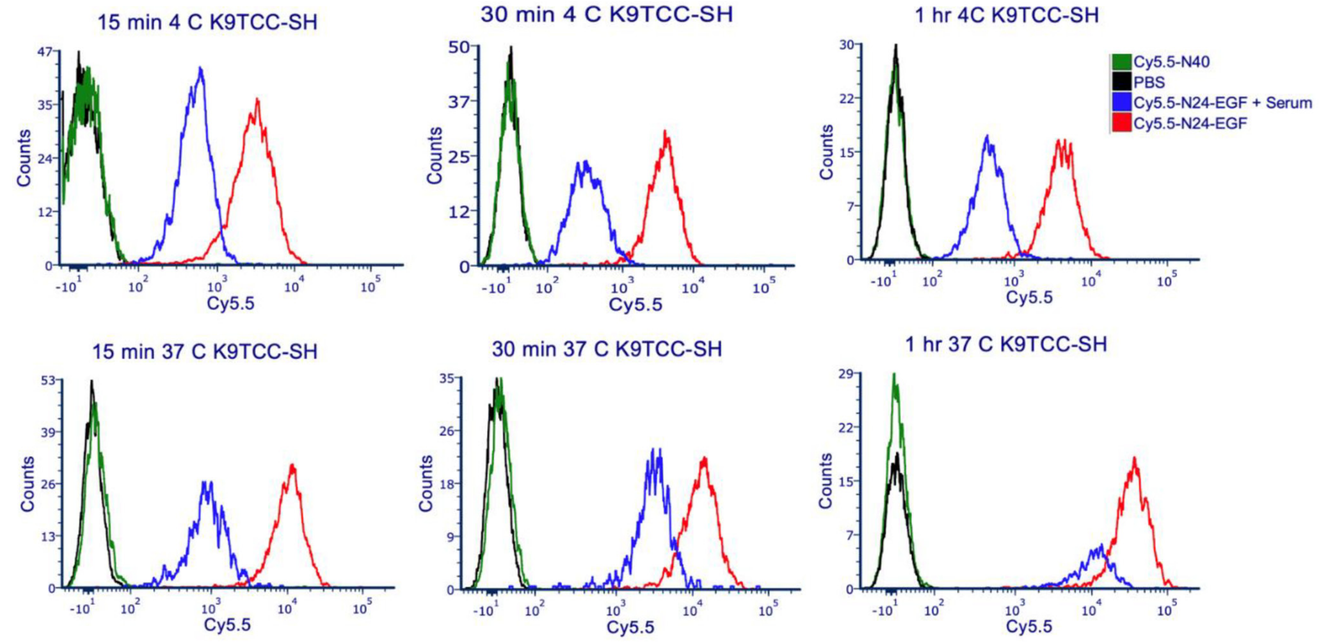

Supplementary Figure 3: Time based binding studies with K9TCC-SH cells at 4°C vs. 37°C to evaluate receptor specific binding. Binding of Cy5.5-N24-EGF to these cells was higher at 37°C than 4°C at all time points. Serum-based competition also had lower binding at 4°C than 37°C for all time points, suggesting reduced EGFR availability on the cell surface.

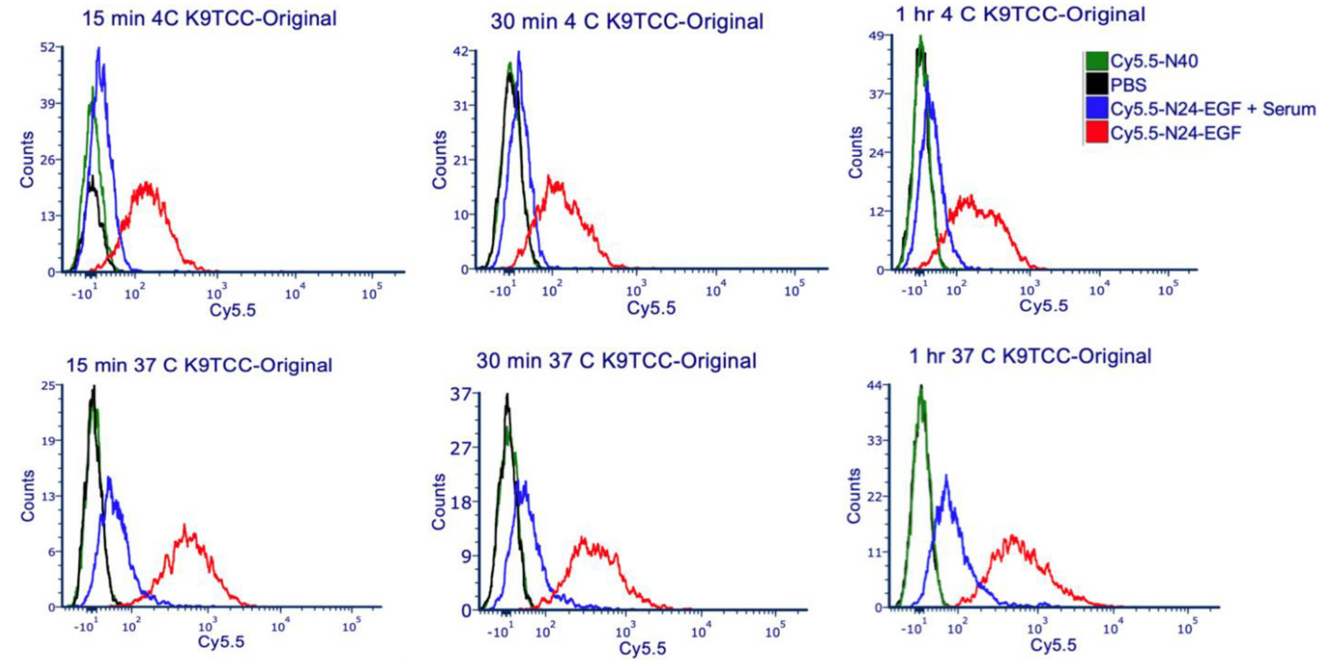

Supplementary Figure 4: Time and temperature based binding studies performed with K9TCC-Original cells at 4°C vs. 37°C for 15 min, 30 min and 1 h to further evaluate EGFR-specific binding. Cy5.5-N24-EGF binding to these cells was higher at 37°C at each time point than for 4°C. Serum-based competition was similar for all time points observed.

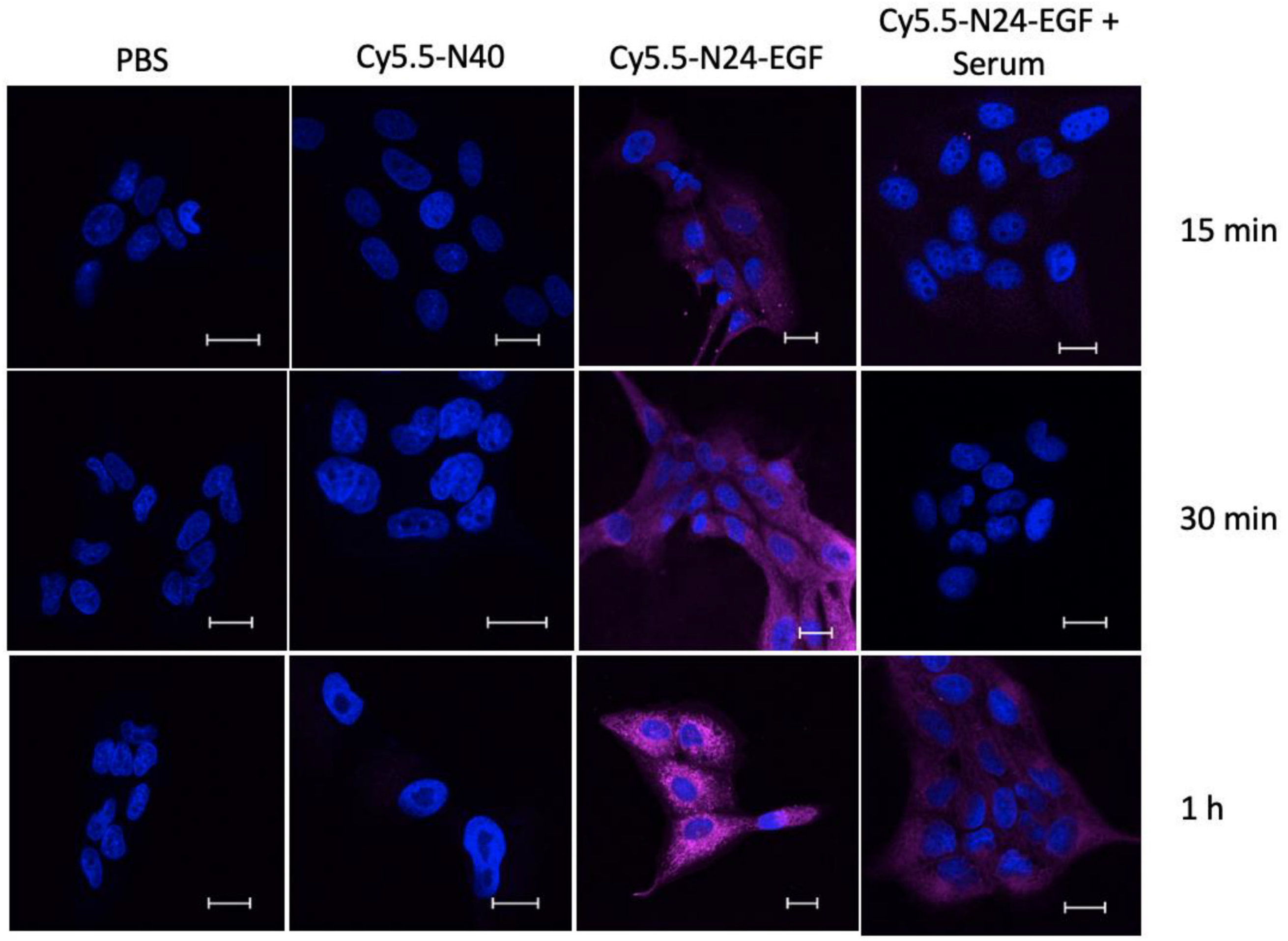

Supplementary Figure 5: Confocal microscopy analysis of time-dependent internalization of probes by K9TCC-SH cells. Samples were imaged using the DAPI (nuclear stain) and Cy5.5 (modified peptide signal) channels. PBS and Cy5.5-N40 revealed no cell-associated Cy5.5 fluorescence. Cells treated with Cy5.5-N24-EGF revealed extensive internalization with an increasing time. Cells treated with Cy5.5-N24-EGF + Serum had minimal signal at 15 min. At 1 h, the Cy5.5 signal for Cy5.5-N24-EGF + Serum increased; however, it was lower than for cells treated with Cy5.5-N24-EGF, in agreement with our flow cytometry findings. Scale bar = 20 µm.

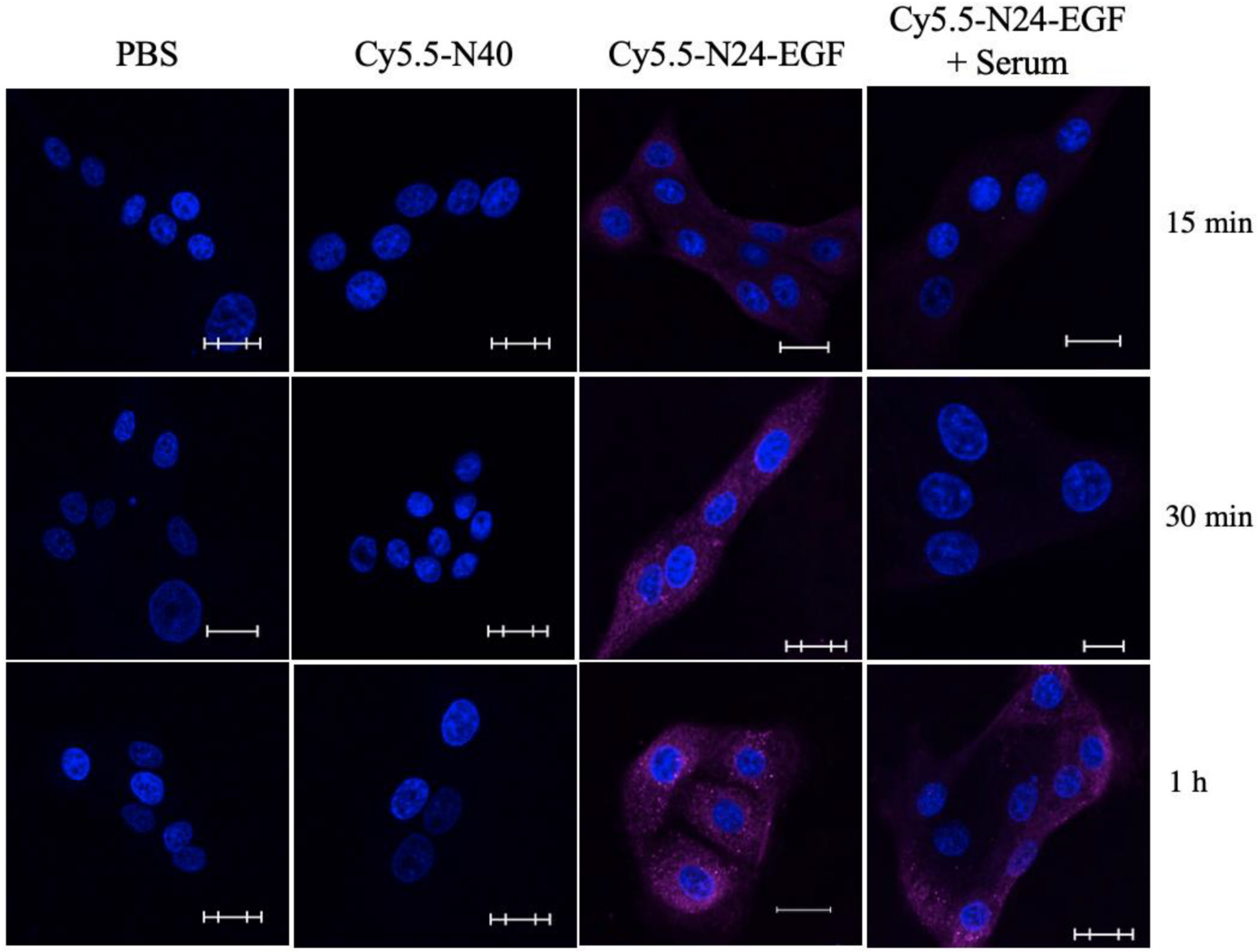

Supplementary Figure 6: Confocal microscopy analysis of time-dependent internalization of probes by K9TCC-Original cells. Samples were imaged using the DAPI (nuclear stain) and Cy5.5 (modified peptide signal) channels. Cells treated with Cy5.5-N40 or PBS produced no detectable Cy5.5 signal. Cells treated with Cy5.5-N24-EGF showed a time-dependent internalization, with the highest Cy5.5 fluorescence intensity observed at 1 h. Cells treated with Cy5.5-N24-EGF + Serum produced a weak Cy5.5 fluorescence at 15 min that gradually increased up to 1 h, in agreement with our flow cytometry findings. Scale bar = 20 μm.

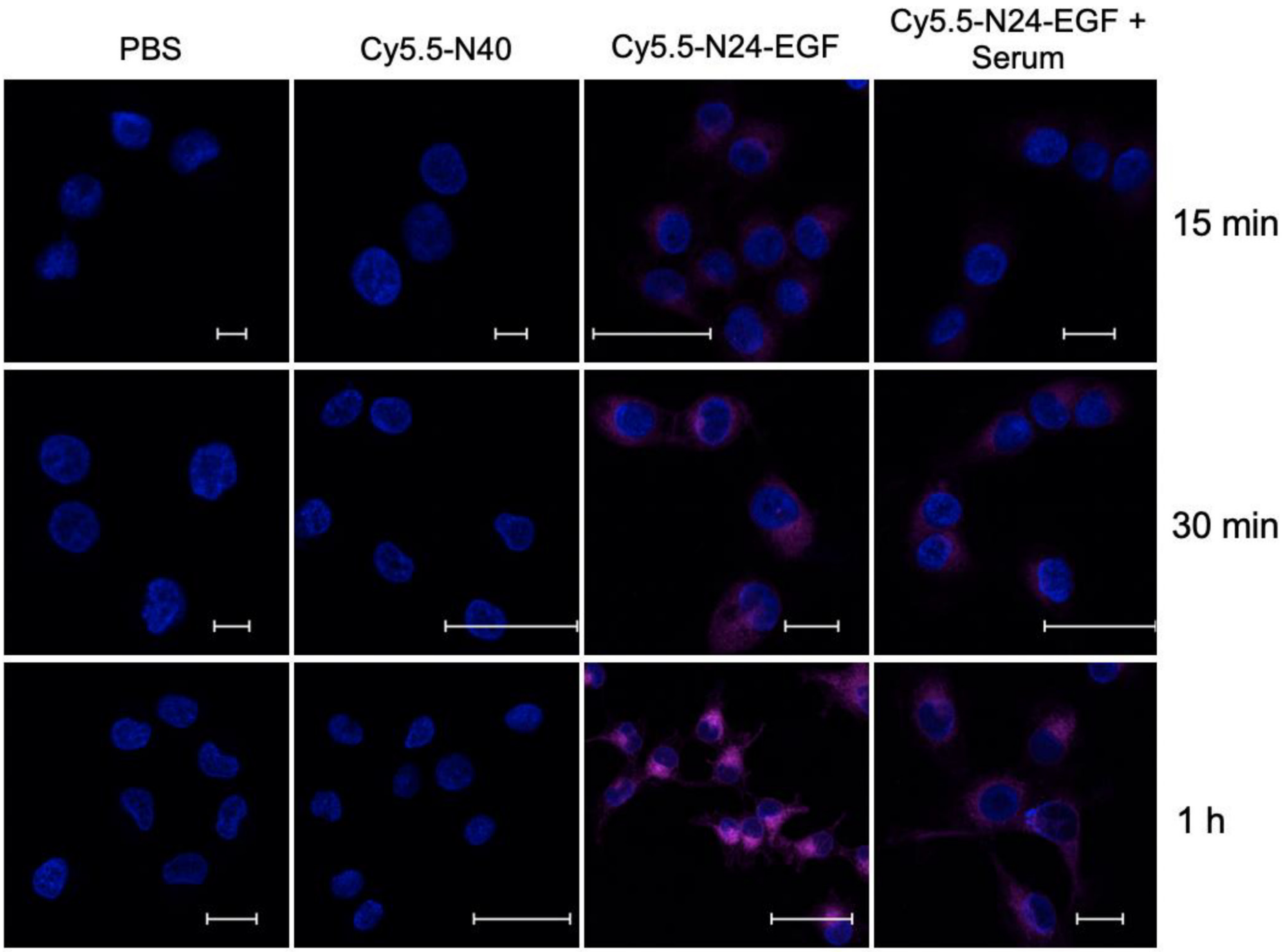

Supplementary Figure 7: Confocal microscopy analysis of time-dependent internalization of probes by T24 cells. Samples were imaged using the DAPI (nuclear stain) and Cy5.5 (modified peptide signal) channels. Cells treated with Cy5.5-N40 or PBS produced no detectable Cy5.5 signal. Cells treated with Cy5.5-N24-EGF displayed internalized Cy5.5 fluorescence within 15 min, with the highest intensity observed at 1 h. Cells treated with Cy5.5-N24-EGF + Serum produced a weak Cy5.5 fluorescence at 30 min that gradually increased up to 1 h, in agreement with our flow cytometry findings. Scale bar = 20 μm.

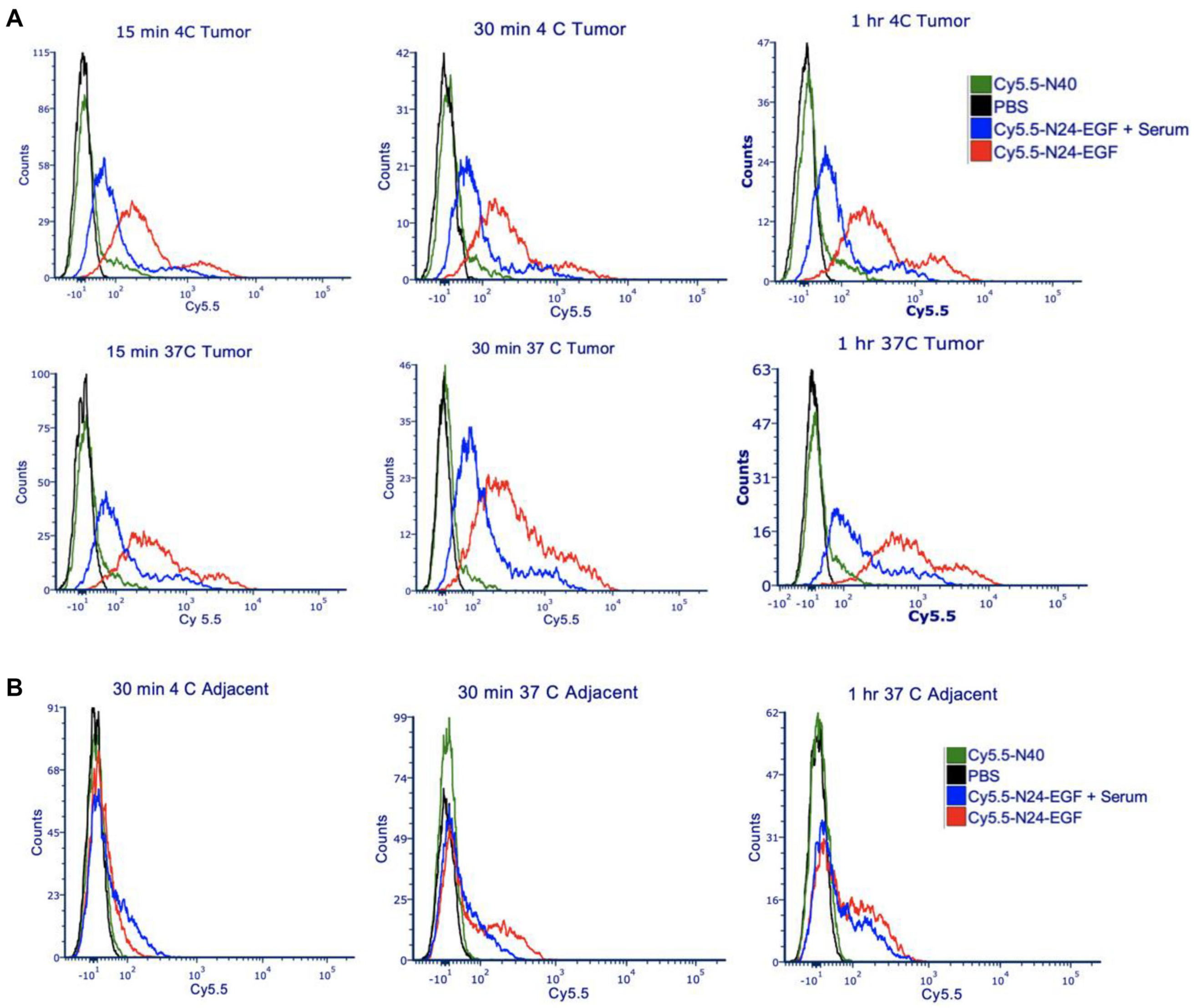

Supplementary Figure 8: *Ex vivo* studies with canine tissues obtained from bladder tumor resection. (**A**) Tumor was homogenized and tested at various times and temperature. The binding of Cy5.5-N24-EGF increased with time relative to samples treated with either Cy5.5-N40 or PBS controls. Incubation at 4°C revealed lower cell associated fluorescence for cells treated with Cy5.5-N24-EGF at 15 min and 30 min comparison to cells incubated with the targeting ligand at 37°C. Competition studies at both temperatures showed decreased cell-associated fluorescence for Cy5.5-N24-EGF, suggesting receptor-specific probe binding *ex vivo*. The non-targeting peptide, Cy5.5-N40 produced little cell-associated fluorescence. (**B**) Adjacent non-tumor tissue was homogenized and treated under the same conditions as those described above. The fluorescence intensities observed were similar at 30 min and 1 h at 37°C for Cy5.5-N24-EGF; serum competition produced a slight reduction at 37°C. The cell-associated fluorescence at all time points for adjacent tissue was much lower than for tumor tissue, indicating higher EGFR density on tumor tissue.

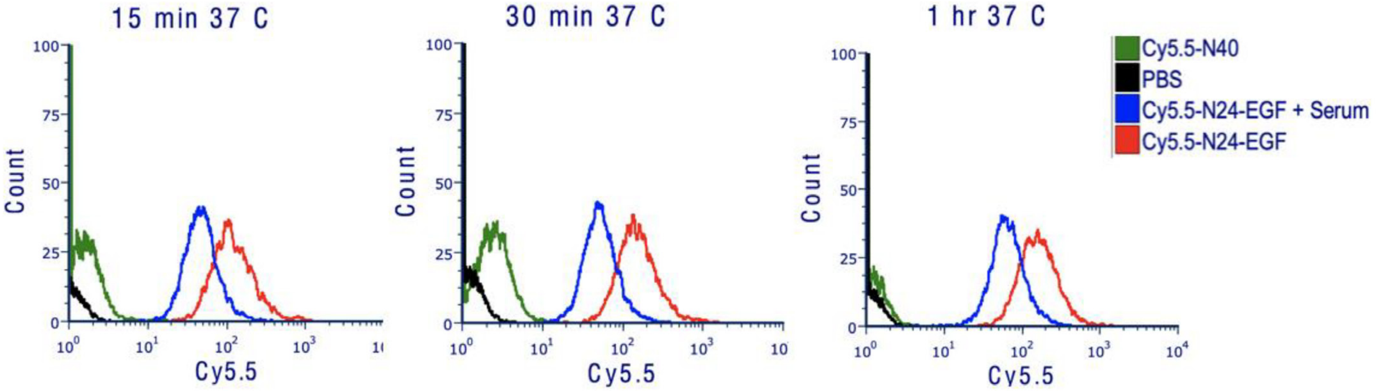

Supplementary Figure 9: Time-dependent binding of probes to cells derived from homogenized human tumor tissue. Cell-associated fluorescence was observed for Cy5.5-N24-EGF within 15 min, with a slight increase observed up to 1 h. Competition experiments with Cy5.5-N24-EGF + Serum produced lower Cy5.5 fluorescence in the cells compared to Cy5.5-N24-EGF at all time points. Minimal cell-associated fluorescence was observed at all time points for samples treated with Cy5.5-N40.

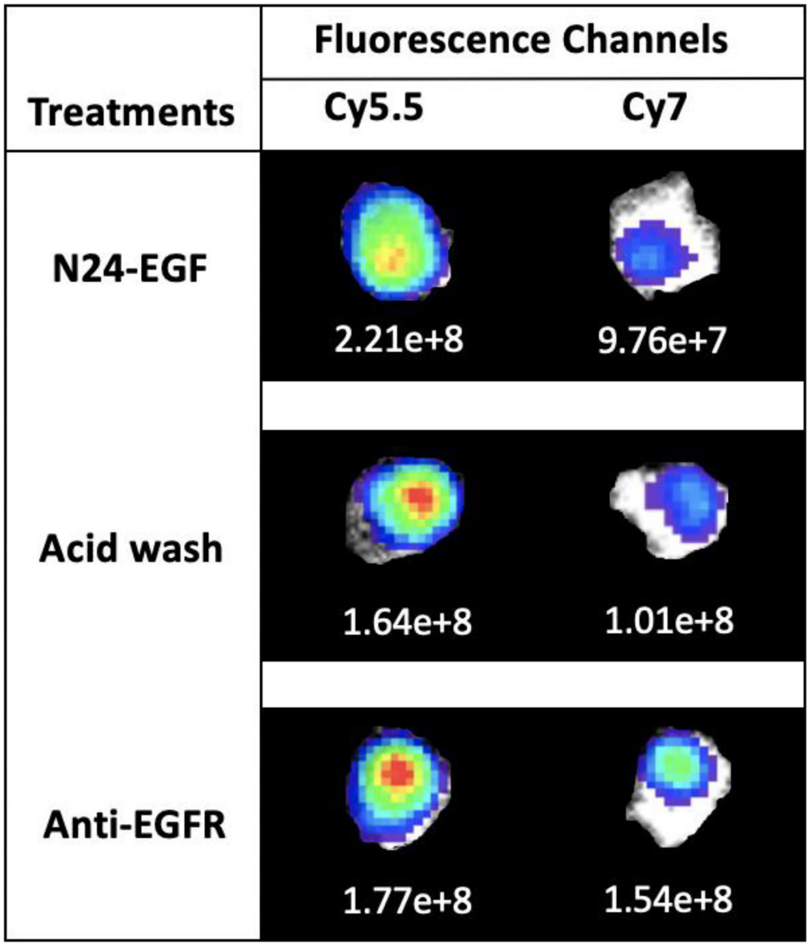

Supplementary Figure 10: Cy5.5-N24-EGF and anti-EGFR staining of samples obtained from the same human tumor sample. The tissue sections were incubated for 30 min at 37°C with Cy5.5-N24-EGF and imaged under Cy5.5 channel. The samples were then washed with acidic buffer to release externally bound Cy5.5-N24-EGF and then re-imaged under Cy5.5 channel to confirm if washing was successful. Finally, PE-Cy7-Anti-EGFR was incubated with the acid washed samples to reveal the presence of surface-available EGFR (same face was analyzed for both anti-EGFR and Cy5.5-N24-EGF associated fluorescence). Increases in fluorescence was observed under Cy7 channel post anti-EGFR staining, due to unblocking of surface EGFR by the bound Cy5.5-N24-EGF ligands.
